# Supplementary material for: A Projection-Domain Low-Count Quantitative SPECT Method for α-Particle-Emitting Radiopharmaceutical Therapy
Source: IEEE Trans Radiat Plasma Med Sci. Author manuscript; Available in PMC 2023 May 17. (PMC10191330; doi:10.1109/trpms.2022.3175435)
Supplement: supp1-3175435 [file NIHMS1861502-supplement-supp1-3175435.pdf]

Supplementary Material

I. Designing, printing, and assembling the vertebrae phantom

In this section, we describe the process to design, print, and assemble the 3D vertebrae phantom that was used as one of the physical phantoms in our studies to validate the proposed low-count quantitative SPECT method.

A. Segmentation and creating 3D models

Anonymized CT scan of an adult male (26 yrs) was used to segment lumbar vertebrae. Using segment editor module of 3D slicer, lumbar vertebrae L1-L5 were segmented. Each vertebra was exported as .stl files for further processing. Blender v2.8 was used to process the 3D models. Operations performed in Blender included cleaning the mesh, fixing normal, retopology, and Laplacian smoothing. After creating clean vertebral models, L4 vertebrae was chosen for creating necessary molds for phantom fabrication. A core for the L4 vertebrae was designed by scaling down the body region of the vertebrae. Thickness of 1 mm was assigned to the designed core. The core was designed to have a hole and a plug to enable refilling and leakproof closing. A shell was designed in two halves around the whole vertebrae with shell thickness of 1 mm.

B. 3D printing

The core and two halves of the mold were 3D printed using FormLabs Form2 3D printer. The core was 3D printed using Clear resin at standard resolution. The mold and plug were printed using Elastic 50A resin at standard resolution. 3D printed parts were post-processed as suggested by the manufacturer. This included support removal, Isopropyl alcohol wash, and curing.

C. Assembling and fabrication of the phantom

The core was placed inside the mold and seams of the molds were taped to hold the parts in place. The void between the core and the mold mimics the cortex of the vertebrae. This void was filled with Plaster of Paris (PoP)(DAP PoP). A slurry of PoP was prepared following the manufacturer's instructions (2 parts powder and 1-part cold water). Using the inlet built into the mold, PoP was poured and let to set overnight. The plaster model was carefully taken out of the elastic mold and spray coated with acrylic paint.

II. Physical phantom preparation

In this section, we describe the process to prepare the two physical phantoms, namely the vertebrae phantom described above and the NEMA phantom, to conduct the physical-phantom-based imaging studies with the goal of evaluating the low-count quantitative SPECT method.

We filled both the phantoms with clinically-relevant $^{223}\mathrm{Ra}$ activity concentrations. $^{223}\mathrm{Ra}$ was purified from a source of Actinium-227 and a source of Thorium-227 for the NEMA and vertebrae phantoms, respectively. We diluted the $^{223}\mathrm{Ra}$ in methanol: nitric acid (2N) (80:20). For each phantom, we prepared the solution of $^{223}\mathrm{Ra}$ with an activity concentration of 40 kBq/ml by diluting with deionized water. The activity was carefully measured with the radionuclide calibrator CRC® -15 Dual PET (Capintec). We filled each sphere of the NEMA phantom with this solution using a 20 ml syringe with an 18Gx6 in needle. The remaining phantom was filled with deionized water to simulate attenuation and scatter due to soft tissue. For the vertebrae phantom, we filled the lesion chamber with the $^{223}\mathrm{Ra}$ solution using a 20 ml syringe with an 18Gx3/4" needle. Then we fixed the filled vertebrae phantom on a cylindrical insert inside the NEMA phantom body. We filled the rest of the phantom with deionized water, modeling soft tissue. This configuration simulated the imaging of a lesion in the spine in the thoracic region.
